# A prospective dual-centre intra-individual controlled study for the treatment of burns comparing dermis graft with split-thickness skin auto-graft

**DOI:** 10.1038/s41598-022-25346-4

**Published:** 2022-12-15

**Authors:** Sinan Dogan, Moustafa Elmasry, Ahmed El-Serafi, Folke Sjöberg, Jyrki Vuola, Esko Kankuri, Marina Perdiki Grigoriadi, Jussi Valtonen, Islam Abdelrahman, Ingrid Steinvall, Matilda Karlsson, Pia Olofsson, Andrew Lindford

**Affiliations:** 1 Department of Hand Surgery, Plastic Surgery and Burns in Linköping, and Department of Biomedical and Clinical Sciences, Linköping University, Linköping, Sweden; 2 Department of Plastic Surgery, Helsinki Burn Centre, Helsinki University Hospital, University of Helsinki, Helsinki, Finland; 3 Department of Pharmacology, Faculty of Medicine, University of Helsinki, Helsinki, Finland; 4 Department of Clinical Pathology, and Department of Biomedical and Clinical Sciences, Linköping University, Linköping, Sweden; 5 Linköping University Hospital, 58185 Linköping, Sweden

**Keywords:** Clinical trials, Randomized controlled trials

## Abstract

To investigate if donor and recipient site morbidity (healing time and cosmesis) could be reduced by a novel, modified split-thickness skin grafting (STSG) technique using a dermal component in the STSG procedure (DG). The STSG technique has been used for 150 years in surgery with limited improvements. Its drawbacks are well known and relate to donor site morbidity and recipient site cosmetic shortcomings (especially mesh patterns, wound contracture, and scarring). The Dermal graft technique (DG) has emerged as an interesting alternative, which reduces donor site morbidity, increases graft yield, and has the potential to avoid the mesh procedure in the STSG procedure due to its elastic properties. A prospective, dual-centre, intra-individual controlled comparison study. Twenty-one patients received both an unmeshed dermis graft and a regular 1:1.5 meshed STSG. Aesthetic and scar assessments were done using The Patient and Observer Scar Assessment Scale (POSAS) and a Cutometer Dual MPA 580 on both donor and recipient sites. These were also examined histologically for remodelling and scar formation. Dermal graft donor sites and the STSG donor sites healed in 8 and 14 days, respectively (*p* < 0.005). Patient-reported POSAS showed better values for colour for all three measurements, i.e., 3, 6, and 12 months, and the observers rated both vascularity and pigmentation better on these occasions (*p* < 0.01). At the recipient site, (n = 21) the mesh patterns were avoided as the DG covered the donor site due to its elastic properties and rendered the meshing procedure unnecessary. Scar formation was seen at the dermal donor and recipient sites after 6 months as in the standard scar healing process. The dermis graft technique, besides potentially rendering a larger graft yield, reduced donor site morbidity, as it healed faster than the standard STSG. Due to its elastic properties, the DG procedure eliminated the meshing requirement (when compared to a 1:1.5 meshed STSG). This promising outcome presented for the DG technique needs to be further explored, especially regarding the elasticity of the dermal graft and its ability to reduce mesh patterns.

*Trial registration*: ClinicalTrials.gov Identifier (NCT05189743) 12/01/2022.

## Introduction

Skin grafting is an important and useful reconstruction option for covering skin defects, irrespective of their cause or site. Amongst skin grafts used for reconstructive surgery, such as split-thickness skin grafts (STSGs), full-thickness skin grafts (FTSGs), free cartilage grafts, and composite grafts; the STSG is the most commonly used and has the broadest application in everyday surgical care^1^. The STSG is defined as a cutaneous free tissue transfer that is intentionally separated from a donor site and transplanted to a recipient site, in which the graft depends on capillary ingrowth for its survival. Although skin grafting has been known for 2500 years, the technique that represents the current STSG technique was first described in 1872 by Ollier de Lyon^2^. This technique has remained essentially the same for 150 years. The technique is far from perfect, and shortcomings relate to donor site morbidity, which has been in focus recently^3^, as well as recipient site scarring. This is a problem well known in the field of burns, for example, where the problem presents not only as a contracture of the scar, but the mesh pattern most often seen post-transplantation contributes especially to non-optimal healing, i.e., scarring. The meshing procedure is regularly undertaken to obtain a graft that covers larger areas than, e.g., the donor site, as the STSG is inelastic and impossible to expand. The meshing procedure is almost always used, except for in the face, where due to cosmetic reasons the STSG graft is unmeshed to avoid the unfavourable mesh pattern and the concomitant risk of worse scarring. A non-meshed STSG graft is called a sheet graft and is well known to improve scar quality at the cost of donor site size.

The use of only the dermis portion as a graft for the recipient site has also been reported in the literature. The skin is then harvested in two layers from the same site, a traditional split-thickness skin graft of the upper layer including the corneal layer of the epidermis, and a dermal graft of the lower layer. The upper part is returned to the donor site, whereas the dermal portion is added to the recipient site. This procedure has gained significant interest lately, as it has favourable characteristics that address the donor site as well as issues at the recipient area in the traditional STSG procedure^4–7^. Especially promising is the fact that the dermal graft is very elastic, and so it may lead to a graft that is expandable. In this case, the need for performing a mesh procedure is reduced or at best avoided.

The aim of this prospective, (intra-individual controlled surgical site), exploratory, dual-centre study was to compare a DG technique with a conventional STSG. The patient group selected for the trial had burns of a limited size. The primary endpoint was donor site morbidity (healing time and scar quality). The secondary exploratory endpoints were: first to examine the dermal graft elasticity and therefore the potential for it to be expanded without a meshing procedure; and secondly to examine the transplant effect of this un-meshed DG at the recipient site (pertaining to healing time and scar quality).

## Methods

### Patients

From February 2017 to December 2020, 23 patients with thermal and chemical burns that fulfilled the eligibility criteria (see below) were consecutively enrolled from the Helsinki Burn Center, Helsinki, Finland and the Linköping Burn Center, Linköping, Sweden.

Inclusion criteria were:

- Thermal & chemical burns < 30% TBSA.
- Males and females between 18 and 80 years.
- At least one coherent full-thickness burn measuring 15 × 7.5 cm.
- A minimum of 15 × 7.5 cm coherent undamaged skin located at the front or lateral side of one of the thighs for graft harvest.

Exclusion criteria were:

- Full-thickness burns of the head, genitalia, axilla, or upper third of the medial upper arm.
- Patients with severe cutaneous trauma at the same site as the burn injury.
- Previous burns at the treatment site.
- Severe cognitive dysfunction or psychiatric disorder.
- Chronic skin disorder.
- Use of systemic or local corticosteroid.
- Use of anticoagulants or platelets.
- Autoimmune disease.
- Active hepatitis.

Two patients died due to severe burn injuries and sepsis and were excluded, leaving a total of 21 included patients. Patients were treated according to a standard burn care protocol in both centres^8–10^.

### Dermal graft and STSG technique

All patients were treated with both methods for skin grafting (STSG and dermis graft). The locations of the skin graft of each method at the donor sites as well as the recipient sites were selected to avoid local heterogeneity in the donor and the recipient site. The latter was the burn injured area, and it was selected if the size was larger than 15 × 7.5 cm and the burn depth was considered not to vary significantly within the area. The areas within the burn wound allocated to the DG or STSG were randomly assigned using closed envelopes just before surgery. The envelopes contained information about where on the wound the dermis graft should be placed and from which part of the donor site (proximal or distal) the graft would be harvested. Intact skin at the thigh was used as a donor site in all patients. The study was approved by the Swedish Ethical Review Authority 2016-401-31.

Initially, an 8/1000 in. (0.203 mm) thick STSG was left attached to one end and folded up after the harvest, and a subsequent 12/1000 in. (0.305 mm) thick dermal split-thickness graft was harvested at the same site using Zimmer Air Dermatome (Zimmer Inc., USA). A surgical assistant aided in the harvest process to maintain tissue tension as well as provide more pressure, which is crucial to get a satisfying DG. To properly harvest a useful DG is a challenge, as it requires training and the extra hands of an assistant. This challenge imposed limitations on the patient recruitment process and was the initial reason why two centres were asked to participate. The Finish centre had already published their experience in the DG procedure^8^.

Adjacent to the DG donor site a 12/1000 in. (0.305 mm) thick STSG graft was harvested according to the standard method. After the harvest, this graft was meshed 1:1.5 using a Zimmer II Mesher.

**Figure 1:**
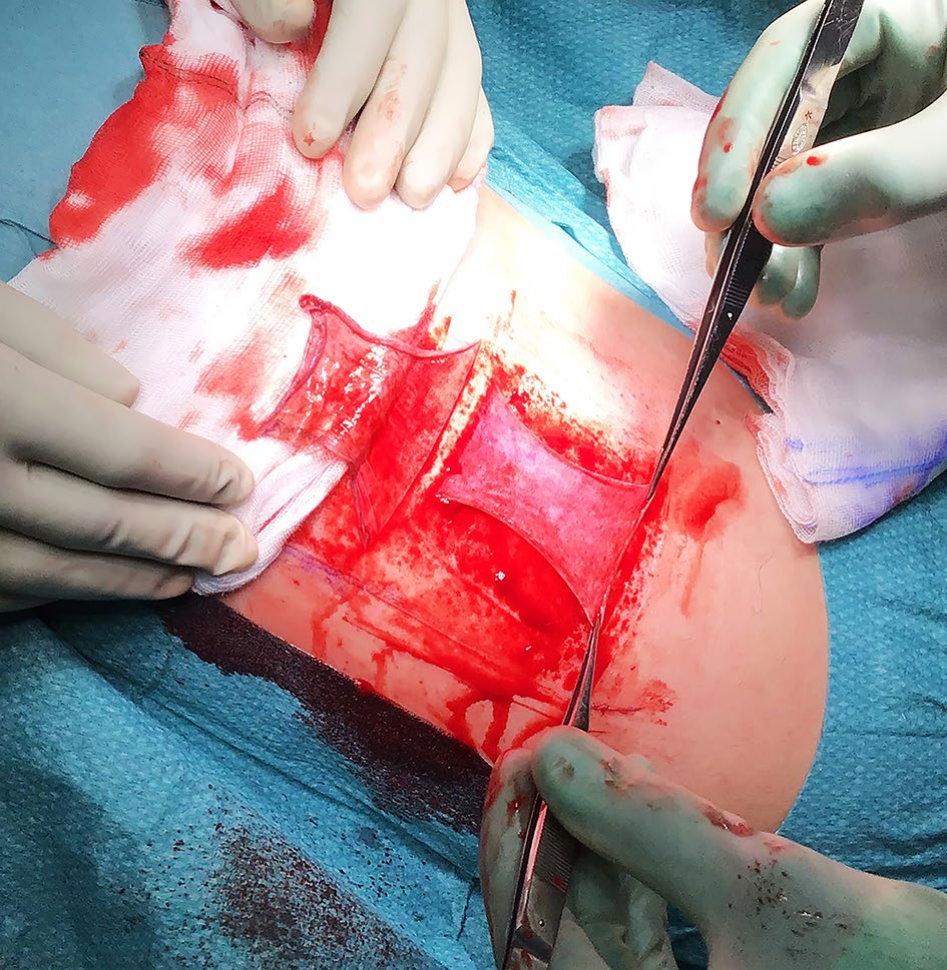
Donor site: STSG graft harvest, folded up (on the cotton swab) and a subsequent split dermal graft harvest (note its elastic consistency).

**Figure 2:**
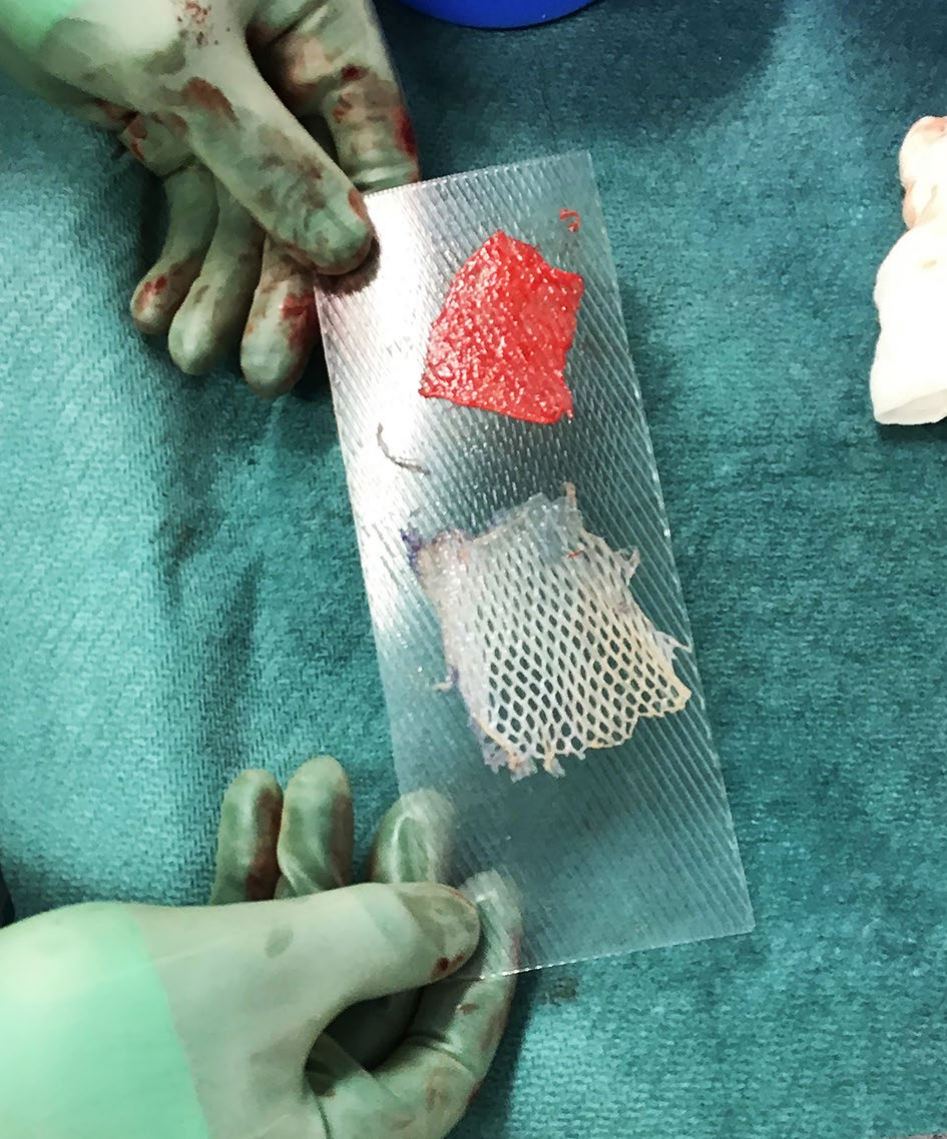
A dermis graft (red) and a meshed STSG graft (white).

**Figure 3:**
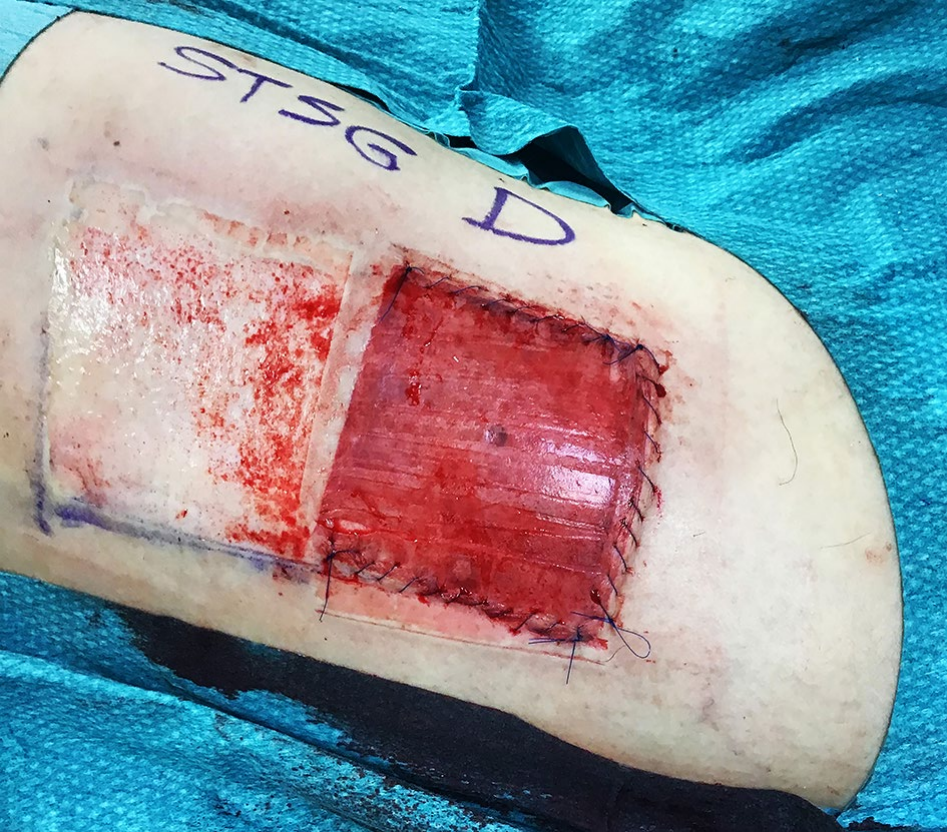
A standard STSG donor site (left) and an adjacent dermal donor site after dermal harvest completion (right).

**Figure 4:**
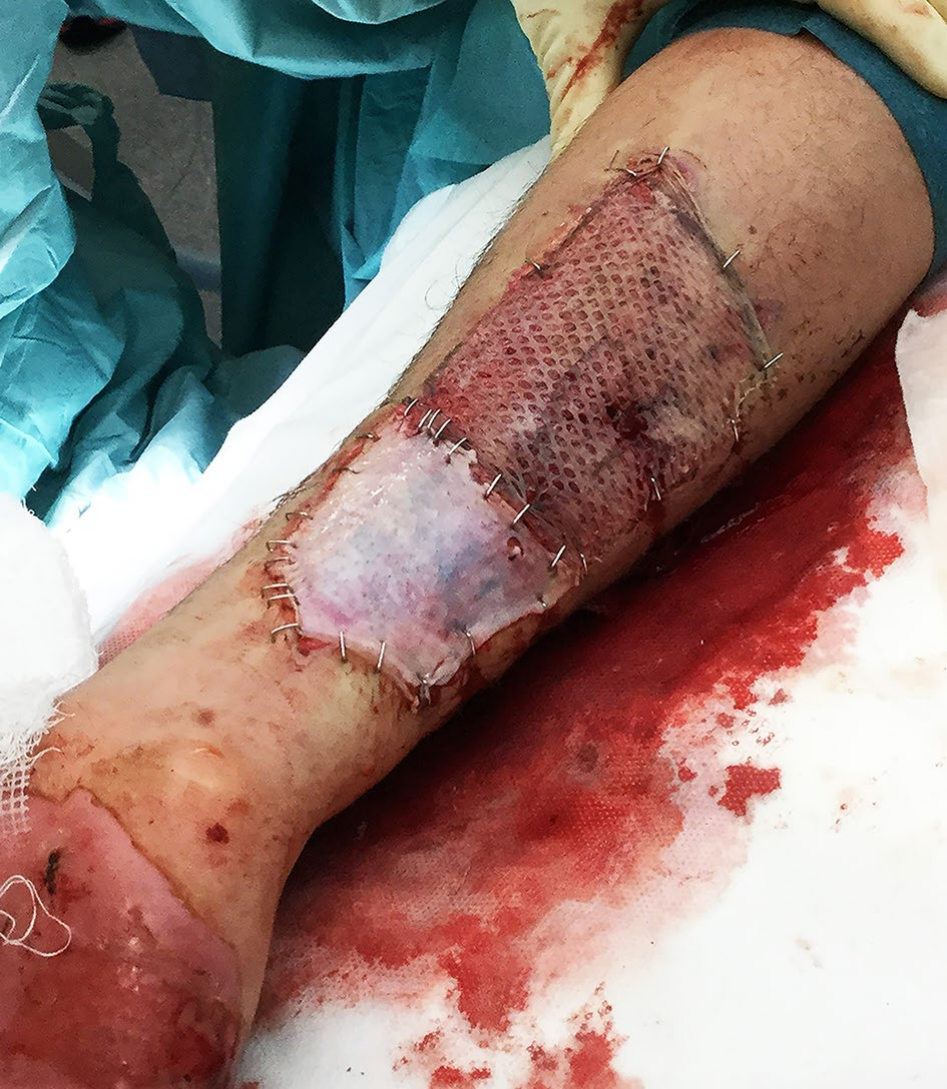
Perioperatively; unmeshed dermal graft (distal) and a meshed STSG graft (proximal) in one of the cases with equal healing time between the two methods.

Directly after surgical debridement, before reinstating the other half of the STSG at the donor site, the donor site was treated with adrenaline-soaked dressings for a minimum of 15 min for haemostatic purposes. The DG was then expanded to cover the excised area and fixed with sutures or staples. Figures 1, 2, 3, and 4 show the whole procedure from graft harvest to coverage of the recipient area.

After haemostasis, the STSG that had been left attached along one edge at the dermal donor site, was reinstated and sutured with a non-absorbable running technique to the wound edges using Prolene® 4-0 (Ethicon Inc.) leaving a covered dermal donor site, and an adjacent standard STSG donor site for comparison (Fig. 3). Figure 4 shows perioperative details of one of the two patients in whom the grafts were placed on an excised area with exposed tendons. In these patients, the dermal graft healed as quickly as the STSG.

Small drainage holes were made to minimise the risk of haematoma underneath the DG. Both donor sites and recipient sites were dressed using conventional paraffin gauze, and finally, pressure was applied using fixed pressure pads and elastic fixation bandage.

A total of four 3 mm punch biopsies were collected, perioperatively and at 6 months’ follow-up, from the dermal and split-thickness graft donor sites as well as the dermal and split-thickness graft recipient sites. The biopsies were fixed in 4% paraformaldehyde, embedded in paraffin, and sectioned at 5 µm. The sections were stained with haematoxylin and eosin as well as Masson’s Trichrome Stain (Sigma-Aldrich, US) and blindly evaluated for histomorphology.

Follow-up healing assessment was done by two independent, experienced burn surgeons. Epithelialisation was considered when > 90% of the wound area had epithelial cover on recipient sites and on the STSG donor site. The healing of the DG donor site was assessed by adherence of the returned skin-flap to the wound bed. Healing of both donor and recipient sites were assessed according to the standard of care within a week after surgical intervention. Healing assessment was done at least twice weekly until completed. Dressings were removed whenever an infection was suspected.

### Scar assessment

Assessment of scar quality and the aesthetic outcome was done by an experienced occupational therapist and a burn surgeon at the trial site using two different reliable and validated tools; The Patient and Observer Scar Assessment Scale (POSAS) and the Cutometer Dual MPA 580^11^. The recipient and donor sites were assessed separately.

The observer scale (objective) of the POSAS consists of six items (vascularity, pigmentation, thickness, relief, pliability, and surface area). The patient scale (subjective) of the POSAS also consists of six items (pain, itching, colour, stiffness, thickness, and irregularity). All items are scored on a scale ranging from 1 (“like normal skin”) to 10 (“worst scar imaginable”) (except for the items pain and itching where the scales go from 1 “no, not at all” to 10 “yes, very much”).

A cutometer was used in this study to assess the viscoelasticity properties of the different grafts and the different donor sites. This was done at the Linköping burn centre only. A 6 mm probe was used, said to best reflect the viscoelasticity of the dermis. A controlled vacuum of 450 mbar was applied in the central area of the scar of interest for two seconds, called “on-time” followed by a relaxation time (with normal pressure) of two seconds called the “off-time”. This was automatically repeated three times, and the results for the corresponding variables R0 and R2 were displayed^12^. Firmness: The R0 variable (U_f_) shows the highest point (maximum amplitude) of the first curve implies the firmness of the skin and represents the passive behaviour of the skin to force. Elasticity: The R2 is calculated by dividing the maximum drop of the curve (U_a_) when removing the vacuum (the relaxation phase) with the R0 value (U_a_/U_f_).

### Data analysis and statistics

Data are presented as median (25th to 75th percentile) or number (in percent) unless otherwise stated. The significance of the difference between variables was assessed with the aid of the Wilcoxon Matched Pair Test. Probabilities of less than 0.05 were accepted as significant.

### Ethics approval and consent to participate

All methods were carried out in accordance with relevant guidelines and regulations. The study was approved by the Swedish Ethical Review Authority 2016-401-31. Informed consent was obtained from all participants.

## Results

### Demographic data

Median age was 53 (44–67) (n = 21) years at admission, 17 (81%) were males. Median (range) percentage total body surface area (TBSA %) burned was 10 (2–16) %. The median (range) duration of hospital stay was 13 (5–30) days, four patients (19%) were treated in the outpatient clinic (minor injuries). The aetiology of the burns were 13 (62%) flame burns, six (29%) contact with hot surfaces, one (5%) chemical burn, and one (5%) scalding. Excision of devitalised tissue was performed a median (range) five (1–11) days after injury. Vast majority of the recipient site was upper and lower extremities, 16 (76%), followed by four (19%) on the abdomen and one (5%) on the back. The excision depth after surgical revision of the wound was recorded on 13 patients and it varied from fascia/muscle (n = 4), subcutis (n = 7) and the deepest layer of dermis (n = 2). Most of the patients were categorised as ASA I or II (Supplemental table S1).

### Surgical outcome

#### Donor site

Dermal graft donor site healing time was shorter, median (range) eight (7–14) days as compared to the STSG donor site; median (range) 14, (12–17) days, (*p* < 0.005) (Supplemental table S1).

#### Recipient site

The DG take (recipient site) was successful in 18 of 21 cases. Two STSG grafts failed due to infection and one because of patient’s poor compliance: In two of the patients a wound infection with *Staphylococcus aureus* and *Pseudomonas aeruginosa* was observed approximately a week after transplantation which resulted in more extensive wound care and delayed healing. All dermis grafts were unmeshed and regarding the STSG grafts, 13 were meshed 1:1.5 before application on the recipient site and these were used for the STSG and DG secondary explorative endpoint evaluation. In eight cases, the wound to be covered by the STSG was smaller and so could be done without meshing, but a few drainage holes were added.

The median (range) healing time of the DG (recipient site) was longer, 22, (14–31) days compared with eight (7–14) days, (*p* < 0.001) of the corresponding STSG graft (Supplemental table S1). Regarding the recipient sites, two dermis grafts had equal healing time as the STSG, and no dermis graft healed before the “corresponding” STSG.

### Scar assessment

**Figure 5:**
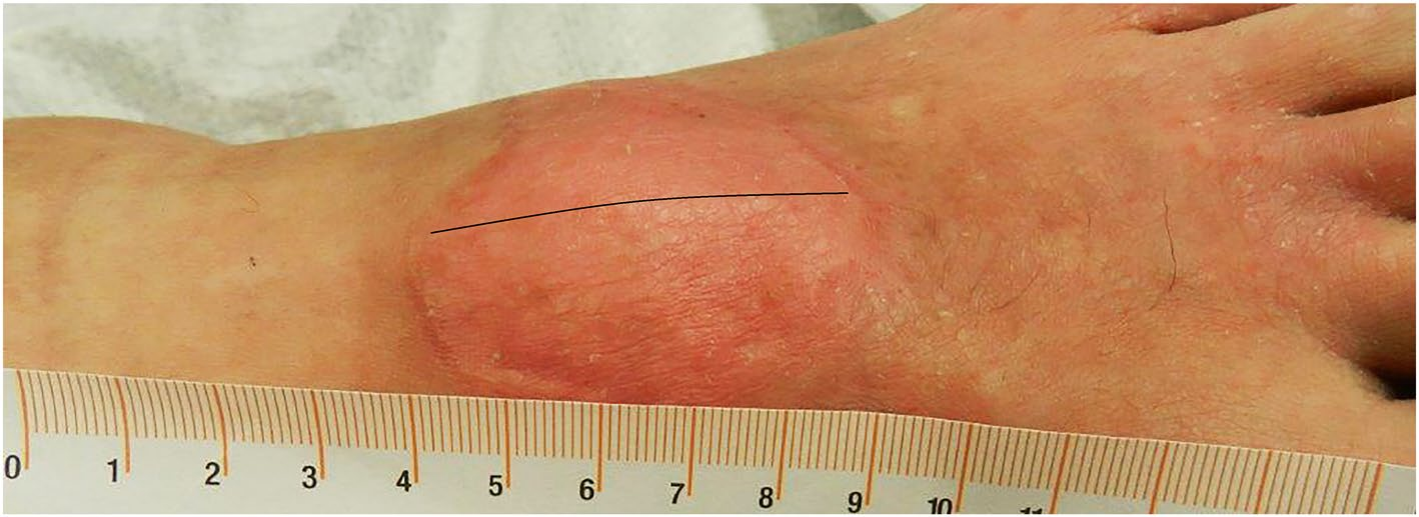
Recipient site follow up at 12 months post-transplantation at left foot. DG site above the black marker (lateral) and STSG site at the bottom (medial).

**Figure 6:**
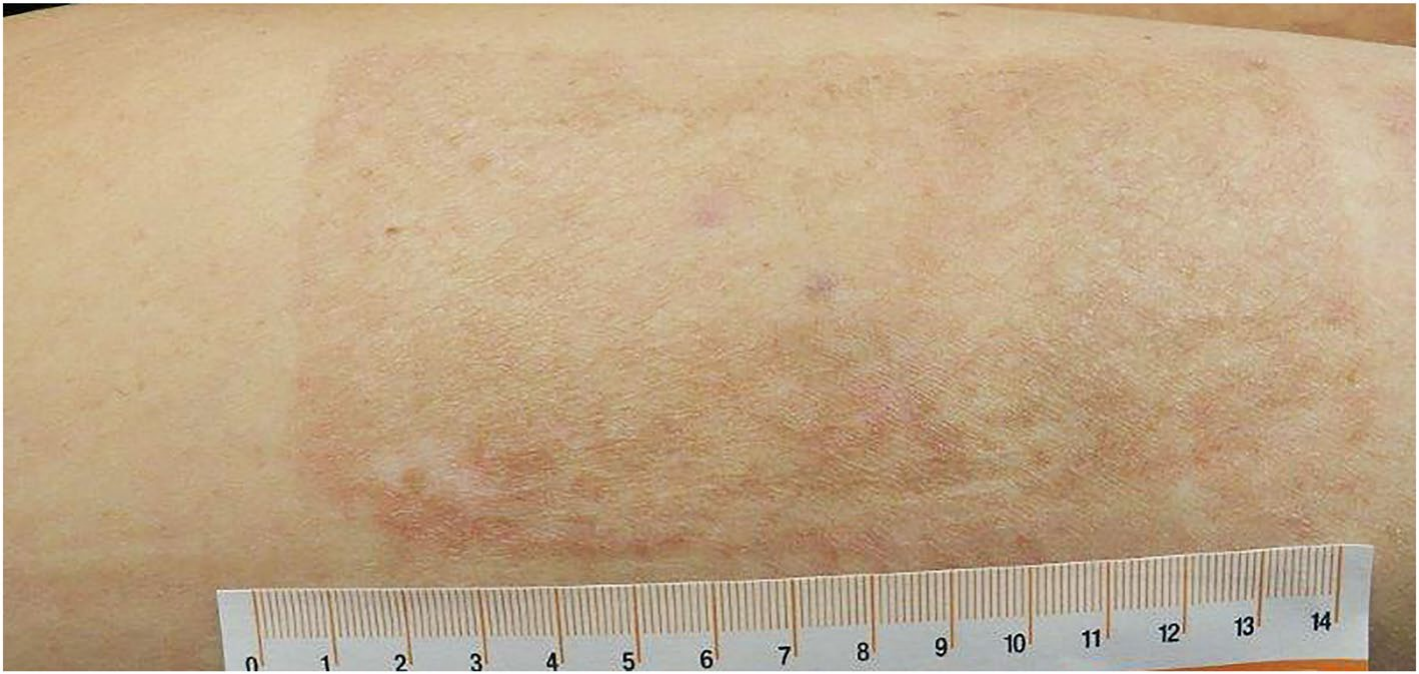
Donor site follow up at 12 months. DG donor site to the right and STSG donor site to the left. The demarcation between the two sites can be seen at the level of 8 cm on the stick.

The scar assessment (POSAS) was done at 3, 6, and 12 months, separately, for the donor and recipient sites. Fifteen (71%), 18 (86%), and 14 (67%) participants had POSAS recordings at the 3, 6, and 12 months follow up, respectively. Figures 5 and 6 show the 12 months’ follow up for both recipient and donor sites.

#### Donor site patient-reported POSAS

The patients reported lower mean POSAS values (better) for colour at all three measurements, the only significant difference was at 6 months (3 months 3.8 (DG) and 4.8 (STSG); 6 months 2.7 (DG) and 3.8 (STSG) (*p* = 0.03) and 12 months 2.9 (DG) and 3.8 (STSG)) (Supplemental table S2). All other outcomes were comparable between DG and STSG.

#### Donor site observer-reported POSAS

The observers rated both vascularity (2.0 (DG) and 2.9 (STSG) at 3 months; 1.8 (DG) and 2.9 (STSG) at 6 months; 1.6 (DG) and 2.4 (STSG) at 12 months, all *p* < 0.01) and pigmentation (2.6 (DG) and 3.0 (STSG) 3 months, 2.2 (DG) and 2.9 (STSG), 1.9 (DG) and 1.9 (STSG)) lower at 3 and 6 months, although not significantly so (Table 1).

**Table 1:** POSAS variables observer—the difference between STSG and DG donor site.

	Dermis graft	STSG	*p*
**3 Months follow up**			
Vascularity	2.0 (0.5)	2.9 (0.9)	0.01
Pigmentation	2.6 (0.9)	3.0 (1.4)	0.15
Thickness	1.6 (0.5)	1.6 (0.6)	1.00
Relief	1.8 (0.7)	1.7 (0.6)	0.53
Pliability	1.6 (0.6)	1.6 (0.6)	0.74
Surface area	4.1 (3.0)	4.4 (3.3)	0.41
**6 Months follow up**			
Vascularity	1.8 (0.4)	2.9 (1.0)	0.01
Pigmentation	2.2 (0.7)	2.9 (1.2)	0.09
Thickness	1.4 (0.5)	1.4 (0.5)	0.69
Relief	1.8 (0.8)	1.4 (0.5)	0.16
Pliability	1.5 (0.5)	1.4 (0.5)	0.36
Surface area	3.6 (2.7)	4.4 (3.5)	0.07
**12 Months follow up**			
Vascularity	1.6 (0.6)	2.4 (0.6)	0.01
Pigmentation	1.9 (0.7)	1.9 (0.7)	1.00
Thickness	1.4 (0.8)	1.3 (0.6)	0.42
Relief	1.7 (0.8)	1.5 (0.9)	0.22
Pliability	1.2 (0.4)	1.1 (0.3)	0.18
Surface area	3.4 (2.7)	3.4 (2.8)	0.48

Data are presented as mean (SD). The *p* values are calculated on the difference between STSG and DG. Wilcoxon Matched Pairs Test. *POSAS* The Patient and Observer Scar Assessment Scale, *STSG* split thickness skin graft, *DG* dermis graft.

#### Recipient site patient-reported POSAS

There were no differences noted in the POSAS evaluation when comparing the DG and STSG grafts at the recipient site. (Supplemental Table S3).

#### Recipient site observer-reported POSAS

The observers reported lower POSAS values for the variable pigmentation at all three occasions (3 months 3.8 (DG) and 4.3 (STSG), 6 months 3.8 (DG) and 4.1 (STSG), 12 months 2.8 (DG) and 3.6 (STSG). Not statistically significantly different but a tendency at 3 and 12 months (*p* = 0.09 and 0.07, respectively) (Table 2).

**Table 2:** POSAS variables observer—the difference between STSG and DG recipient site.

	Dermis graft	STSG	*p*
**3 Months follow up**			
Vascularity	4.4 (1.6)	3.3 (1.1)	0.051
Pigmentation	3.8 (1.5)	4.3 (1.7)	0.09
Thickness	3.4 (1.5)	3.3 (1.4)	0.80
Relief	3.3 (2.1)	3.5 (2.0)	0.72
Pliability	3.7 (1.7)	3.4 (1.5)	0.36
Surface area	5.7 (2.8)	5.6 (2.8)	0.69
**6 Months follow up**			
Vascularity	4.0 (2.0)	3.4 (2.4)	0.14
Pigmentation	3.8 (2.0)	4.1 (1.7)	0.51
Thickness	3.2 (1.6)	3.2 (1.8)	0.89
Relief	3.4 (1.3)	3.4 (1.8)	0.69
Pliability	3.3 (1.7)	3.4 (1.9)	1.00
Surface area	5.1 (3.0)	5.2 (2.2)	0.79
**12 Months follow up**			
Vascularity	2.5 (1.0)	2.6 (1.8)	0.67
Pigmentation	2.8 (0.9)	3.6 (1.7)	0.07
Thickness	2.4 (1.1)	2.6 (1.8)	0.92
Relief	2.3 (0.9)	2.8 (1.7)	0.31
Pliability	2.5 (1.1)	2.9 (1.8)	0.53
Surface area	5.1 (3.2)	5.4 (3.3)	0.87

Data are presented as mean (SD). The *p* values are calculated on the difference between split thickness skin graft (STSG) and dermis graft (DG). Wilcoxon Matched Pairs Test. *POSAS* The Patient and Observer Scar Assessment Scale.

### Viscoelasticity properties of recipient and donor sites

Of the 13 study participants at the burn centre in Linköping nine were assessed at the 3 months’ follow up, six at the 6 months’ follow up and seven at the 12 months’ follow up. Eight of the participants had more than one follow up with the Cutometer. The dermal graft at the recipient site was more elastic than the STSG at the 6 months’ follow up (mean (SD) R2 was 0.85 (0.08) and 0.74 (0.11), respectively, *p* = 0.008). Other than that, we found no differences in the viscoelastic properties between the different grafts and the different donor sites. The analysis of differences in viscoelasticity over time in grafts and donor sites showed that the STSG became less elastic between 3 and 6 months (mean (SD) R2 decreased from 0.88 (0.07) to 0.74 (0.11), *p* = 0.04), and that the dermal grafts became less firm between 3 and 12 months (mean (SD) R0 increased from 0.59 (0.26) to 0.82 (0.25), *p* = 0.04), and less elastic between six and 12 months (mean (SD) R2 decreased from 0.85 (0.08) to 0.79 (0.13), *p* = 0.01). (Supplemental Table S4).

### Histological analysis

The histological analysis was blinded to the pathologist. The microscopic architecture was similar between the donor and recipient sites. The histological evaluation of the biopsies confirmed skin healing, irrespective of the biopsy site. The epidermal thickness was slightly larger in the dermal recipient site (median 124 µm and IQR 102–247) in comparison to the split-thickness recipient site (112 µm and IQR 79–128, *p* = 0.07) although not significantly so. The dermal donor site thickness was 99 µm with IQR 73–117, *p* = 0.87). The split thickness donor site was 100 µm and IQR 87–114, *p* = 0.87).

**Figure 7:**
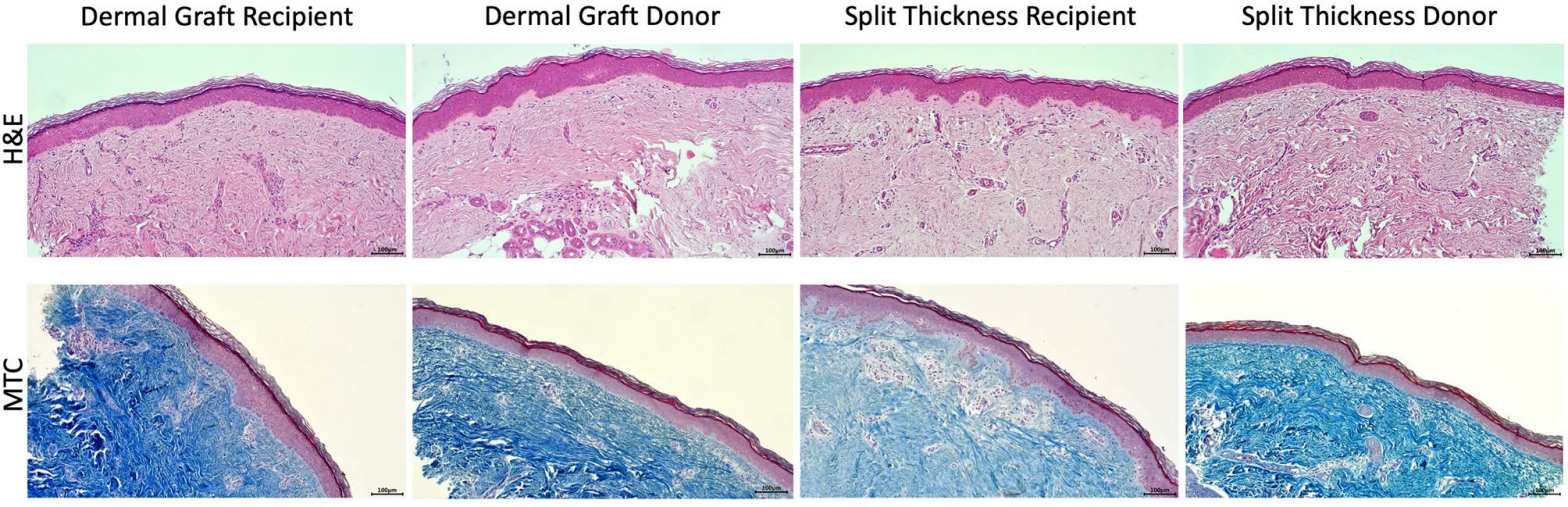
Sections from the four study sites at 6 months were stained with haematoxylin and eosin (H&E) as well as Masson’s trichrome stain (MTC). The skin multilayer structure was preserved in all sites. Collagen deposition was slightly larger in the dermal graft recipient site as noted by the dense blue staining with MTC in the dermis.

The multilayer arrangement was comparable across the four locations with the tendency of a larger collagen deposition in the dermal graft recipient site. (Fig. 7).

## Discussion

The new and important findings in this prospective, dual-centre, intra-individual, controlled (within patient donor/recipient site) study are that healing time and cosmesis were improved at the donor site, findings that have been suggested previously in uncontrolled case series^13,14^. The potential for minimising donor site morbidity is very interesting and for certain groups of patients (such as children, the elderly, or patients with scarring tendency or diminished wound healing potential due to comorbidities) this could be of real benefit.

Further, during the study, it was appreciated early that the elasticity of the non-meshed DG was considerable, reducing the need for a mesh procedure. The result of which may be important for the future, as the mesh pattern after “meshed” regular STSG procedure is well-known to negatively affect post-surgical cosmesis^15,16^. Other indications of improved viscoelastic effects at the dermal graft recipient site in this study were the tendency to a better elasticity as noted by cutometer measurements. This improved elasticity measure was also supported by the more pronounced collagen deposition seen in the histopathological examinations. However, these findings, especially at the recipient site, need to be further examined as the cutometer assessments were only made at one site (Sweden) and patients were lost at follow up. The explorative finding at the recipient site further suggests, in line with the finding at the donor site, that pigmentation also appeared better, as judged by POSAS (observer), but the findings did not reach statistical significance. The healing time was significantly longer for the DG at the recipient site, similar to our previous study^8^. This may be due to the thickness chosen for the DG and can probably be shortened using a thinner DG. Another explanation would be that the dermal graft needs time to vascularise prior to epithelialisation, which then results in a prolonged healing time. Other attempts, such as negative pressure treatment, may be tried to reduce the healing time at the recipient site and are needed to further optimise this new approach to skin grafting. Other options would be covering the dermal graft with an epidermal skin substitute such as Suprathel ® (PolyMedics Innovations GmbH, Denkendorf, Germany)^17^, cell suspension therapy^18,19^ allograft skin, or another novel skin grafting technique^20^.

The previous experience of using a strict and isolated dermal component in the STSG technique was first introduced by Hynes already in 1954, as an alternative to the direct or tubed flap^21^. After this attempt, two publications described a modified technique by which the dermal graft was applied upside down at the recipient site^22,23^. The graft was thereafter left to vascularise and as it did not epithelialise at the surface, a second STSG was applied on top after 2 weeks. It needs to be stressed that the STSG taken at the donor site at the time of the dermal graft was returned to its original position in our study. In the publication by Querings et al. in 2002, it was emphasised that both the functional and cosmetic results at the recipient site were favourable^23^.

Tanabe et al.^7^ used the dermal grafting technique to reconstruct palmar skin defects of the digits and hand using plantar dermal grafts. The dermal graft healed in 12–16 days when there was a good wound bed. In this case series of 17 patients, there was excellent graft take (99.1%), with favourable cosmetic results and no marginal scarring. The STSG to the donor sites healed in 7–8 days with no marginal scarring and good cosmetic results. Histological observation 1 year after dermal grafting, the stratum lucidum had been remodelled and sweat gland tissues were observed^7^.

An experimental study by Rubis et al.^6^ in 2002, in a pig model, they used two layers of dermal grafts and concluded that the epithelialisation was slower at the dermal recipient site than the donor site as there is a need for vascularisation of the graft prior to epithelialisation. Despite this, all grafts were healed in 2 weeks (90% area covered) and the cosmetic results were good.

Another attempt to increase the availability of autologous skin, by use of dermal grafts in the treatment of burns was the study by Kogan et al.^13^. It was concluded that in all eight patients included in the study, there were good dermal grafts “take” with rapid or slow epithelialisation.

The largest study yet, using dermal graft for wound coverage, is the study by Han et al.^4^. They compared dermal grafting to STSG in a plastic surgery setting. The wounds of the dermis (n = 53) and skin grafts (n = 33) had re-epithelialised after 15 and 12 days, respectively. Scarring on the recipient site of the dermis graft was found to be less severe than that on the regular skin graft in terms of pigmentation, height, and vascularity (Vancouver scar scale). It was concluded that the dermal grafting technique was superior both aesthetically and functionally to the regular skin grafting technique in both the recipient and donor sites^4^. These findings are similar to ours.

In the dermal graft study in the burn setting performed by the senior author, 16 dermal grafts performed in nine patients were compared to regular STSG. As a control donor site (STSG) an adjacent area on the back close to the dermal graft donor was selected. The focus of this study was primarily to generate more autologous skin for burn-injured patients. Time to dermis graft epithelialisation ranged from 12 to 35 days (median 21 days). All grafts recorded had > 90% epithelialisation by 4 weeks. There was no significant difference in donor site healing times when comparing the dermal graft “deeper” donor site (range 7–35 days, mean 16.1 days) to the standard control SSG donor site (range 7–35 days, mean 16.7 days)^8^.

In a small (n = 4) patient case series by Kang et al., it was shown that dermal grafts may be successfully applied on top of exposed bone and tendons^24^. Han et al. further demonstrated, in a retrospective study, the satisfying cosmetic as a functional outcome of the dermis graft after coverage of small skin defects postoperatively after tumour resection involving the face^25^. Most of the patients had satisfactory results in both functional and cosmetic matters with high-quality skin characteristics. Patients’ satisfaction with the dermis graft was also excellent.

The strength of this investigation is that it is, to the best of our knowledge, the first dual-centre, prospective, intra-individual, controlled, (within patient, two treatments) study of dermal grafting to examine both donor and recipient site morbidity and cosmesis as assessed by both observer and patient (POSAS). The evaluation tools used, the POSAS instrument, and the cutometer are well-known and properly validated instruments. Although the difference was small, as can be seen in the figures, it was measurable by the POSAS. Histological analysis was also performed. The small study population in the present study is a significant short coming and to detect aesthetic differences, especially at the recipient site, further studies with larger samples sizes will be required. The viscoelasticity measurements and biopsy follow ups were not investigated on all patients due to missed appointments and follow up. Another limitation is that the meshing procedure of the STSG was different between the centres (13 meshed 8 unmeshed), which can have affected our results. However, considering that the aesthetic result likely would be better with an unmeshed STSG the advantage of the DG may have been more pronounced if all STSG had been meshed.

The technique is new and there is no established instrument yet for the dermal graft harvest. Therefore, significant training is required to get satisfying grafts yield with a regular dermatome and this contributed to the small sample size. The technique of taking a DG is more demanding technically compared to a standard STSG harvest. The first layer, the STSG, is usually managed rather easily by a trained plastic surgeon. When taking the second layer, the DG, it needs to be appreciated that the tissue is much less firm and as such poses a significant challenge. The challenges can be summarised by: the lack of firmness which makes it difficult to ascertain the thickness of the graft; and secondly, to harvest a proper DG the procedure needs to be adjusted to the four borders of the first STSG. The research group has, for the future development of this technique, developed a new dermatome that simultaneously collects two layers of skin in a pre-set fashion. We hope that this will be advantageous for further studies as it will make the technical learning curve of the DG procedure less steep, and as the method becomes less challenging it becomes more established and it will be easier to recruit patient for future studies. This technical issue significantly contributed to the challenges of including patients. The inter-rater reliability was not evaluated, which may affect the results in a multicentre study with several investigators. The POSAS reviewers were not blinded towards which site received which graft type, which could have introduced bias. However, despite this, the POSAS instrument has been claimed to be adequate.

Additionally, regarding the elasticity of the DG, it was appreciated early during the study that the DG had a prominent elasticity, not necessitating a meshing procedure to cover the larger recipient areas. It is a significant limitation of the present protocol that we cannot present data on the expansion rate of the DG. This is an important topic to address for the next study. However, we think that the fact that the unmeshed DG could be expanded so that it covered the recipient area of a similar size as the meshed STSG supports the idea of the potential of a favourable cosmetic outcome for the recipient site of the DG as compared to a regular STSG.

## Conclusion

The dermis graft reduced donor site morbidity, because returning the STSG component to the donor area heals faster than the standard STSG technique. The long-term outcomes of the recipient sites were also favourable because the elasticity of the dermal graft facilitated the graft expansion without necessitating the mesh procedure. An inherent advantage of the dermal graft technique (not examined in this study) is the double or triple graft yield that can be accomplished to cover large burn areas. However, the strength of the conclusions in this study is hampered by the limited number of patients, the loss of observations between sites, and patient drop-out at follow up. Larger studies should therefore be initiated to explore the advantages and disadvantages of the technique, particularly at the recipient site.

## Supplementary Information

Supplementary Information.

## Data Availability

The dataset used and/or analysed during the current study are available from the corresponding author on reasonable request.
