# Supplementary material for: A prospective dual-centre intra-individual controlled study for the treatment of burns comparing dermis graft with split-thickness skin auto-graft
Source: Sci Rep. 2022 Dec 15;12:21666. doi: 10.1038/s41598-022-25346-4 (PMC9755129; doi:10.1038/s41598-022-25346-4)
Supplement: Supplementary file 1 — Supplementary Information. [file 41598_2022_25346_MOESM1_ESM.pdf]

A prospective dual-centre intra-individual controlled study for the treatment of burns comparing dermis graft with split-thickness skin auto-graft

Sinan Dogan, MD[1*], Moustafa Elmasry, MD PhD[1], Ahmed El-Serafi MD PhD[1], Folke Sjöberg, MD PhD[1], Jyrki Vuola, MD PhD[2], Esko Kankuri, MD PhD[3], Marina Perdiki Grigoriadi, MD PhD[4], Jussi Valtonen MD PhD[2], Islam Abdelrahman, MD, PhD[1], Ingrid Steinvall, PhD[1], Matilda Karlsson, PhD[1], Pia Olofsson, MD PhD[1], Andrew Lindford, MD PhD[2]

Supplemental Table S1 – Details of the patients

		p
Age, years	53.0 (44.0–67.0)	
Sex, male	17 (81)	
ASA class 1/2/3	10/2/1	
Burn Size, TBSA %	10.0 (2.0–16.0)	
Duration of hospital stay, days	13 (5.0–30.0)	
Burn type		
Flame	13 (62)	
Contact sauna	6 (29)	
Chemical	1 (5)	
Scald	1 (5)	
Location of recipient		
Upper extremity	8 (38)	
Lower extremity	8 (38)	
Abdomen	4 (19)	
Back	1 (5)	
Excision post injury, days	5.0 (1.0–11.0)	
Excision depth post revision*		
Dermis	2 (15)	
Subcutis	7 (50)	
Fascia, muscle	4 (27)	
Healing STSG donor, days	14.0 (12.0–17.0)	0.005
Healing DG donor, days	8.0 (7.0–14.0)	
Healing STSG recipient, days	8.0 (7.0–14.0)	<0.001
Healing DG recipient, days	22.0 (14.0–31.0)	

Data are presented as median (25th - 75th centiles) or n (%). The p values are calculated on the difference between split thickness skin graft (STSG) and dermis graft (DG). (Wilcoxon Matched Pairs Test). TBSA = total body surface area. *Missing data on excision depth post revision n=8.

Supplemental TableS2. POSAS variables Patient reported – The difference between STSG and DG donor site

	Dermis graft	STSG	p
3 Month follow up			
Pain	1.6 (2.0)	1.2 (0.6)	1.00
Itching	3.3 (2.6)	2.7 (1.8)	0.25
Color	3.8 (2.7)	4.8 (2.4)	0.19
Stiffness	2.6 (1.9)	2.7 (2.3)	0.89
Thickness	1.9 (2.0)	1.9 (1.5)	0.89
Irregularity	3.1 (3.3)	2.3 (1.5)	0.27
6 Month follow up			
Pain	1.2 (0.6)	1.1 (0.3)	1.00
Itching	1.4 (0.9)	1.2 (0.4)	0.18
Color	2.7 (1.4)	3.8 (2.5)	0.03
Stiffness	1.8 (1.3)	1.6 (0.9)	0.65
Thickness	1.7 (1.2)	1.8 (1.5)	0.18
Irregularity	1.8 (1.2)	1.5 (0.8)	0.27
12 Month follow up			
Pain	1.1 (0.3)	1.6 (1.9)	0.18
Itching	1.4 (0.8)	1.4 (0.8)	1.00
Color	2.9 (2.8)	3.8 (2.9)	0.13
Stiffness	1.8 (1.4)	1.6 (1.3)	0.42
Thickness	1.8 (1.9)	2.1 (2.0)	0.42
Irregularity	2.1 (2.3)	1.9 (1.9)	0.18

Data are presented as mean (SD). The p values are calculated on the difference between split thickness skin graft (STSG) and dermis graft (DG). Wilcoxon Matched Pairs Test.

Supplemental Table S3. POSAS variables Patient reported – The difference between STSG and DG Recipient site

	Dermis graft	STSG	p
3 Month follow up			
Pain	3.0 (2.4)	2.5 (2.4)	0.46
Itching	4.7 (2.8)	4.3 (2.7)	0.46
Color	7.5 (1.9)	6.5 (2.6)	0.11
Stiffness	5.5 (2.0)	5.3 (2.0)	0.96
Thickness	5.4 (2.9)	5.3 (2.5)	0.61
Irregularity	5.5 (2.7)	5.3 (2.1)	0.67
6 Month follow up			
Pain	2.5 (2.4)	2.4 (1.9)	0.79
Itching	2.8 (2.6)	3.1 (2.6)	0.36
Color	5.6 (1.6)	5.2 (2.4)	0.84
Stiffness	4.5 (2.1)	3.7 (2.4)	0.39
Thickness	3.5 (2.0)	3.5 (2.1)	0.86
Irregularity	4.4 (2.1)	4.2 (2.4)	0.58
12 Month follow up			
Pain	1.4 (1.1)	2.4 (2.5)	0.14
Itching	2.7 (2.8)	2.7 (2.5)	1.00
Color	5.1 (2.9)	5.1 (3.3)	0.61
Stiffness	3.7 (3.2)	3.9 (3.2)	0.39
Thickness	4.2 (3.4)	4.1 (3.2)	0.94
Irregularity	3.8 (3.2)	4.4 (3.0)	0.23

Data are presented as mean (SD). The p values are calculated on the difference between STSG and DG. Wilcoxon Matched Pairs Test. POSAS = The Patient and Observer Scar Assessment Scale. STSG = split thickness skin graft. DG = dermis graft.

Supplemental Table S4 – Viscoelastic properties of the two treatment arms, Cutometer values of grafts and donor sites at the follow ups

	3 months (n=9)		6 months (n=6)		12 months (n=7)	
	R0	R2	R0	R2	R0	R2
Recipient site						
STSG	0.62 (0.19)	0.88 (0.07)	0.69 (0.11)	0.74 (0.11)	0.63 (0.22)	0.79 (0.06)
DG	0.59 (0.26)	0.98 (0.39)	0.59 (0.10)	0.85 (0.08)	0.82 (0.25)	0.79 (0.13)
Donor site						
STSG	1.01 (0.19)	0.86 (0.12)	1.16 (0.17)	0.84 (0.10)	1.08 (0.20)	0.84 (0.10)
DG	0.90 (0.16)	0.85 (0.06)	0.98 (0.24)	0.89 (0.06)	0.99 (0.10)	0.84 (0.08)

Data are mean and standard deviation in brackets. R0 values (firmness) are given in millimetres and R2 values (elasticity) as quotient.

STSG = split thickness skin graft. DG = dermis graft.
